# Silicon Enhances Water Stress Tolerance by Improving Root Hydraulic Conductance in *Solanum lycopersicum* L.

**DOI:** 10.3389/fpls.2016.00196

**Published:** 2016-02-22

**Authors:** Yu Shi, Yi Zhang, Weihua Han, Ru Feng, Yanhong Hu, Jia Guo, Haijun Gong

**Affiliations:** ^1^College of Horticulture, Northwest A&F UniversityYangling, China; ^2^College of Horticulture, Shanxi Agricultural UniversityTaigu, China

**Keywords:** tomato (*Solanum lycopersicum*), water stress, silicon (Si), root hydraulic conductance, oxidative damage

## Abstract

Silicon (Si) can improve drought tolerance in plants, but the mechanism is still not fully understood. Previous research has been concentrating on Si’s role in leaf water maintenance in Si accumulators, while little information is available on its role in water uptake and in less Si-accumulating plants. Here, we investigated the effects of Si on root water uptake and its role in decreasing oxidative damage in relation to root hydraulic conductance in tomato (*Solanum lycopersicum* ‘Zhongza No.9’) under water stress. Tomato seedlings were subjected to water stress induced by 10% (w/v) polyethylene glycol-6000 in the absence or presence of 2.5 mM added silicate. The results showed that Si addition ameliorated the inhibition in tomato growth and photosynthesis, and improved water status under water stress. The root hydraulic conductance of tomato plants was decreased under water stress, and it was significantly increased by added Si. There was no significant contribution of osmotic adjustment in Si-enhanced root water uptake under water stress. The transcriptions of plasma membrane aquaporin genes were not obviously changed by Si under water stress. Water stress increased the production of reactive oxygen species and induced oxidative damage, while added Si reversed these. In addition, Si addition increased the activities of superoxide dismutase and catalase and the levels of ascorbic acid and glutathione in the roots under stress. It is concluded that Si enhances the water stress tolerance via enhancing root hydraulic conductance and water uptake in tomato plants. Si-mediated decrease in membrane oxidative damage may have contributed to the enhanced root hydraulic conductance.

## Introduction

Silicon (Si) is the second most abundant element in the earth’s crust after oxygen (Zhu and Gong, 2014). It is taken up in the form of silicic acid by plants and is the only nutrient element that is not detrimental when accumulated excessively in plants (Ma et al., 2001). The beneficial roles of Si in combating various biotic and abiotic stresses have been widely reported (Van Bockhaven et al., 2013; Zhu and Gong, 2014). The alleviative effects of Si under drought/water stress conditions have been observed in some Si-accumulating plant species, such as rice (Ming et al., 2012), maize (Kaya et al., 2006), wheat (Pei et al., 2010; Gong and Chen, 2012), and sorghum (Liu et al., 2014).

Previous researchers have explored the mechanisms for Si-mediated drought tolerance. It has been proposed that Si can enhance antioxidant defense and thus decrease oxidative stress in plants under drought (Gong et al., 2005, 2008). Si addition can increase the photosynthesis and relevant carboxylase activities under field drought conditions, as observed in wheat (Gong and Chen, 2012). In maize, Si addition can increase the levels of K and Ca (Kaya et al., 2006), suggesting an important role of Si in keeping the mineral balance in plants.

The positive role of Si in plant water conservation has already been studied (see review by Zhu and Gong, 2014). It has been suggested that Si deposition in the rice leaves can decrease the transpiration via cuticle (Matoh et al., 1991) and thus improve drought tolerance. However, in maize, Si addition does not affect the leaf cuticular transpiration, but significantly decreases the stomatal transpiration (Gao et al., 2006). Nevertheless, these studies suggest that the decrease in leaf transpiration is an important mechanism for Si-mediated drought tolerance. However, in some cases, Si addition does not decrease the transpiration rate of plants under drought, while the plant water status is better in Si-added plants (Gong et al., 2005; Hattori et al., 2005). These observations suggest that Si-mediated increase in drought tolerance may be not only associated with leaf transpiration, but also associated with root water uptake. However, the role of Si in regulating root water uptake has long been ignored. Until recently, Liu et al. (2014) investigated the effect of Si on water uptake and transport in sorghum seedlings under water stress. They found that Si addition increased the root hydraulic conductance, and the increase was attributed to Si-mediated transcription up-regulation of some aquaporin genes.

Although quite some work has been done to explore the mechanism for Si-mediated drought tolerance, yet most of the previous research was conducted on Si-accumulating plant species (Zhu and Gong, 2014). In these gramineous plants, the mechanical/physical barrier induced by Si deposition on plant surface may have contributed to the observed drought tolerance directly or indirectly. Up to date, however, little research has been conducted on dicotyledonous plants, which usually have low capabilities of Si accumulation. Studying the role of Si in low Si-accumulating plants will help to clarify the biochemical function of Si and understand the exact mechanisms for Si-mediated drought tolerance in plants, rather than mere mechanical/physical barrier induced by Si deposition in Si-accumulating plants. As pointed out by Katz (2014) recently, the effect of Si is not proportional to its accumulation in plants, and that low Si accumulation does not mean its low function. Therefore, more work is needed to clarify the function of Si in low Si-accumulating plants.

Tomato (*Solanum lycopersicum* L.) has become a model organism in plant genetics and stress resistance research, both for applied and theoretical purposes (Bergougnoux, 2014). Compared with the typical Si accumulators such as rice and wheat, tomato has much less Si accumulation and has been classified as a ‘Si excluder’ (Nikolic et al., 2007). Therefore, tomato is an ideal model to explore the possible biochemical mechanism for Si-mediated stress tolerance. The positive effect of Si on salt tolerance of tomato has been reported (Muneer et al., 2014; Muneer and Jeong, 2015). However, there have been few reports about the possible role of Si on tolerance of tomato under drought/water stress conditions.

In plants, the limiting factor of water transport is mainly in the roots (Rubio-Asensio et al., 2014). Root hydraulic conductance (Lp) is an important parameter representing the water uptake capacity (Liu et al., 2014). The amount and activity of water channels, which are known as aquaporins in cellular membranes, play key roles in regulating root water uptake, especially under stress conditions (Liu et al., 2014). In addition, osmotic gradient can also facilitate root water uptake (Javot and Maurel, 2002) and therefore contribute to the increase of Lp. The activities of aquaporins are affected by multiple factors, such as ABA, Ca^2+^, and reactive oxygen species (ROS; Liu et al., 2015). ROS has been found to be able to regulate the aquaporin activities by oxidant gating or inducing its internalization (Ye and Steudle, 2006; Boursiac et al., 2008a,b). Recently, Liu et al. (2015) found that Si addition increased the root hydraulic conductance of sorghum (a “silicon-accumulator”) under salt stress and suggested that this was attributed to the expression up-regulation of plasma membrane aquaporins and their enhanced activities by reducing H_2_O_2_ accumulation. However, little information is available about this possible relationship between Si-mediated changes in root hydraulic conductance and H_2_O_2_ accumulation under water stress. It would also be interesting to investigate this relationship in less Si-accumulating plants.

In this work, the effects of Si on plant water status and root water uptake, and the role of Si in decreasing oxidative damage were investigated in tomato seedlings under water stress. Our results showed a positive effect of Si on water stress tolerance in tomato plants, and suggest that Si increased water stress tolerance by decreasing oxidative damage and enhancing root hydraulic conductance. Our work in tomato – a “silicon-excluder” may help to better understand the mechanism for Si-mediated water stress/drought tolerance in plants.

## Materials and Methods

### Plant Materials and Treatments

Seeds of tomato (*Solanum lycopersicum* ‘Zhongza No.9’) were sterilized in 55°C water bath for 25 min, immersed in distilled water for 6 h, germinated on two layers of moist filter paper for 2 days in an incubator at 28°C, and then sown in washed commix medium (Xintiandi Co., Yangling, Shaanxi, China). At four-leaf stage, the seedlings were transplanted into plastic boxes, each of which contained 12 L of half-strength Hoagland solution (pH 6.2) in an environmentally controlled glasshouse, and maintained at 25–30°C during the day and 15–18°C at night, with 60–75% of relative humidity.

At five-leaf stage, the seedlings were treated with different solutions as follows: (1) CT = 1/2 Hoagland solution without addition of Si or polyethylene glycol (PEG); (2) Si = 1/2 Hoagland solution with addition of 2.5 mM Si; (3) PEG = 1/2 Hoagland solution with addition of 10% (w/v) PEG-6000; and (4) PEG + Si = 1/2 Hoagland solution with addition of 10% (w/v) PEG-6000 and 2.5 mM Si. PEG-6000 was used to induce water deficit stress. Potassium silicate (K_2_SiO_3_) was used as the Si source, and preliminary experiments showed that 2.5 mM was a suitable silicon concentration. The introduced potassium due to K_2_SiO_3_ addition was subtracted from potassium nitrate, and the resultant loss of nitrate ions was supplemented with dilute nitric acid. The solution pH was adjusted to 6.2 daily. The treatments lasted for 7 days. All the experiments were repeated at least three times.

### Photosynthetic Parameters and Biomass Determination

Photosynthetic gas exchange parameters were measured on the second recently fully expanded leaves with a LI-6400 portable photosynthesis system (LI-COR Inc., USA) at 9:00–11:00 am after 5 days of water stress. The measurements were conducted with the photosynthetic active radiation of 800 μmol m^-2^ s^-1^.

For biomass assay, after 7 days of water stress treatment, the plants were collected, washed with distilled water, dried in an oven at 75°C for 72 h, and weighed.

### Determination of Leaf Relative Water Content and Water Content

The relative water content and water content were determined on the second recently fully expanded leaves after 7 days of water stress according to Ming et al. (2012). The leaf water potential was measured by a pressure chamber (PMS 1505D, USA) according to Yin et al. (2013).

### Determination of Root Hydraulic Conductance

The root hydraulic conductance (Lp) was measured after 5 and 7 days of stress by a pressure chamber (PMS 1505D, USA) according to the method of Miyamoto et al. (2001).

### Determination of Root Osmotic Potential and Proline Concentration

Tomato root osmotic potential (Ψ_π_) and proline concentration were determined after 5 and 7 days of water stress. The osmotic potential was measured according to Yin et al. (2013) using a vapor pressure osmometer (Model 5520, Wescor, Logan, UT, USA). The osmotic potential was calculated according to the formula: Ψ_π_ = –RTC, where R, T, and C were molar gas constant, thermodynamic temperature and osmolality (mmol kg^-1^) from the osmometer, respectively.

The proline content was determined using the method of Bates et al. (1973). Briefly, the samples were extracted in 3% sulfosalicylic acid in boiling water, and the proline content was assayed using the ninhydrin regent.

### Expression Analysis of Aquaporin Genes

The roots of tomato seedlings were collected and frozen in liquid nitrogen, and immediately stored at –80°C until analysis. The sampling time was 8 h, 1, 3, 5, and 7 days. Total RNA was extracted from 0.1 g of root samples using an RNeasy Plant Mini Kit (Tiangen DP419, Beijing, China) according to the manufacturer’s instructions. The first-strand cDNA for qPCR analysis was synthesized from 1 μg of total RNA using a PrimeScript^TM^ RT reagent Kit (Takara Bio, Shiga, Japan) according to the manufacturer’s instructions, including a special step to remove the remaining genomic DNA. The plasma membrane gene-specific primers were designed and listed in **Table 1** and the qPCR reaction was conducted on a Bio-Rad CFX-96 real-time PCR system (Bio-Rad, USA) using SYBR^®^ Premix Ex Taq^TM^ (Takara Bio, Shiga, Japan). The relative transcript levels of target genes were normalized to that of the internal control actin gene using the 2^-ΔΔCT^ method (Pfaﬄ, 2001). Each treatment included three replications.

**Table 1:** Gene specific primers used for real-time PCR.

Gene	Locus	Primer Sequence
*SlPIP1;3*	Solyc12g056220.1	F: 5′-AGCTCCTCTGTTTGAACCA-3′
		R: 5′-TACCACCAAAAGCCCAAGCAA-3′
*SlPIP1;5*	Solyc08g081190.2	F: 5′-CAGCTCCATTGTTTGAACCAG-3′
		R: 5′-TCATACCACCAAAAGCCCAA-3′
*SlPIP2;6*	Solyc11g069430.1	F: 5′-TTAAGGCTTTTCAAAGTGCAT-3′
		R: 5′-CGGAGAAGACAACATAGACC-3′
*Actin*	X55749	F: 5′-GATGGTGTCAGCCACAC-3′
		R: 5′-ATTCCAGCAGCTTCCATTCC-3′

*F-forward primer; R-reverse primer.*

### Determination of Relative Electrolyte Leakage

The relative electrolyte leakage of roots was measured after 5 days of water stress treatment according to Wang et al. (2013) using an electrical conductivity meter.

### Determination of Malondialdehyde Content

The malondialdehyde contents in roots were dynamically monitored (1, 3, 5, and 7days) based on the thiobarbituric acid reaction (Bailly et al., 1996) with slight modifications. Root tissues (0.5 g each) were homogenized in 8 ml of 0.1% (w/v) trichloroacetic acid and the homogenates were centrifuged at 4,830 × *g* for 10 min at 4°C, after which the supernatants were used for malondialdehyde analysis. Equal volumes of extracts were mixed with 0.5% (w/v) of thiobarbituric acid made in 5% (w/v) trichloroacetic acid and heated at 100°C water bath for 20 min, after which the reactions were stopped in ice bath. After centrifuging at 7,888 × *g* for 10 min, the absorbance of the supernatant was measured at 450, 532, and 600 nm. The malondialdehyde content was calculated using the formula: malondialdehyde content (μmol g^-1^ DW) = [6.452 (OD_532_ – OD_600_) – 0.559 × OD_450_] × 8/(DW × 1.5).

### Determination of Reactive Oxygen Species (ROS) Level

The ROS levels were monitored after 5 and 7 days of water stress. The $O_{2}^{•-}$ production rates in roots were assayed according the method of Elstner and Heupel (1976). The H_2_O_2_ contents in roots were assayed according to the method of Csiszár et al. (2012).

### Histochemical Staining Analysis

The histochemical staining analyses were conducted after 6 days of water stress. The plasma membrane integrity of roots was monitored by the Evans blue staining method according to Wang and Yang (2005). The roots were incubated in 0.025% (w/v) Evans blue solution for 30 min, and then washed with distilled water three times, after which the roots were observed under a light microscope (BX51, Olympus, Japan) and photographed. The levels of superoxide anion radical ($O_{2}^{•-}$) and hydrogen peroxide (H_2_O_2_) were examined according to Xu et al. (2012). The membrane lipid peroxidation damage was measured using the Schiff’s reagent staining method according to Pompella et al. (1987).

### Determination of Antioxidant Enzyme Activity and Antioxidant Substance Content

The antioxidant enzyme activity and antioxidant substance content in roots were dynamically monitored (1, 3, 5, and 7 days). The antioxidant enzymes were extracted according to Gong et al. (2005) and the supernatant was used to determine the activities of superoxide dismutase (SOD) and catalase (CAT). SOD activity was assayed by the nitroblue tetrazolium method according to Gong et al. (2005). CAT activity was measured according to Yang et al. (2010).

The concentrations of reduced glutathione (GSH) were assayed according to the method of Paradiso et al. (2008). The ascorbic acid (AsA) contents were assayed according to Huang et al. (2005).

### Statistical Analysis

Data were subjected to One-way ANOVA (analysis of variance) with SAS software (SAS Institute, Cary, NC, USA). Where *F* tests were significant (*P* < 0.05), means were separated by Duncan’s multiple range test.

## Results

### Plant Growth and Photosynthesis

**FIGURE 1:**
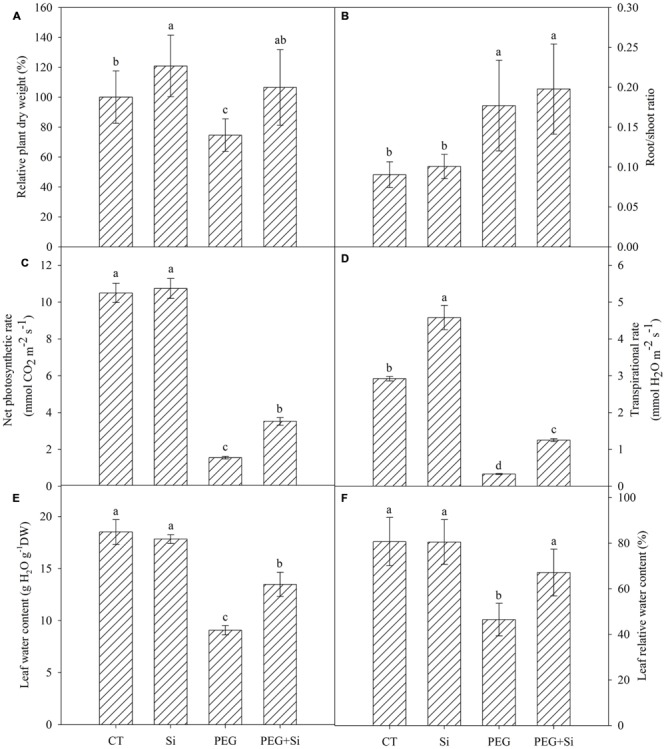
**Effects of silicon on the relative plant dry weight, photosynthetic gas exchange and leaf water status of tomato seedlings grown under polyethylene glycol-simulated water deficit stress.** Data are shown as means ± SD. The plant dry weight was measured after 7 days of water stress, and the pooled data were from three independent culture experiments (*n* = 11). The photosynthetic gas exchange measurement was conducted on the second fully expanded leaves (from top) 5 days after water stress (*n* = 4). The leaf water content and relative water content were measured on the second fully expanded leaves (from top) 7 days after water stress (*n* = 3). Bars with the same letters are not significantly different at *P* < 0.05. CT, control; DW, dry weight; Si, silicon; PEG, polyethylene glycol-induced water stress; PEG + Si, PEG plus silicon. **(A)** Relative plant dry weight; **(B)** Root/shoot ratio; **(C)** Net photosynthetic rate; **(D)** Transpirational rate; **(E)** Leaf water content; **(F)** Leaf relative water content.

Under non-stress conditions, added Si slightly increased the plant dry weight (**Figure 1A**). The dry weight was significantly decreased under water stress, and it was higher in Si-added stressed plants. In non-stress conditions, Si addition did not change the net photosynthetic rate or transpirational rate (**Figures 1C,D**). Under water stress, the net photosynthetic rate and transpirational rate were both decreased significantly, and they were maintained higher in Si-added stressed plants (**Figures 1C,D**).

### Plant Water Status

Under control conditions, Si addition did not change the water status in the leaves (**Figures 1E,F**). Under water stress, the leaf relative water content and water content were significantly decreased, while addition of Si partially reduced the decrease (**Figures 1E,F**).

### Root Hydraulic Conductance

**FIGURE 2:**
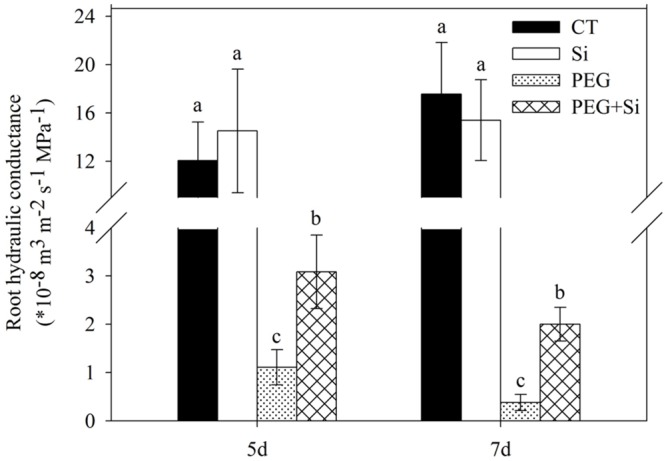
**Effects of silicon on root hydraulic conductivity of tomato seedlings grown under water stress.** The measurements were conducted 5 and 7 days after water stress. Data are shown as means ± SD (*n* = 5). Bars with the same letters are not significantly different at *P* < 0.05.

As shown in **Figure 2**, added Si did not affect the root hydraulic conductance of tomato seedlings under non-stress conditions. The root hydraulic conductance was dramatically decreased under water stress, and it was maintained significantly higher in Si-added stressed plants.

### Root Osmotic Potential and Proline Concentration

**FIGURE 3:**
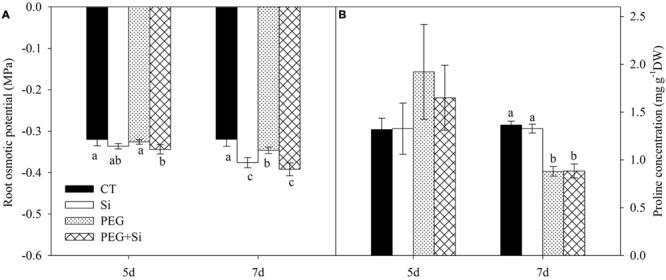
**Effects of silicon on root osmotic potential and proline concentration of tomato seedlings grown under water stress.** Data are means ± SD (*n* = 4 for osmotic potential, and 3 for proline concentrations). Bars without letters or with the same letters are not significantly different between treatments at *P* < 0.05 at each time point. **(A)** Root osmotic potential; **(B)** Proline concentration.

In non-stress conditions, the root osmotic potential was not significantly changed by Si treatment on the fifth day, but it was significantly increased after 7 days of Si treatment (**Figure 3A**). Under water stress, the root osmotic potential was not changed on the fifth day, but it was slightly decreased on the seventh day; and silicon-added plants had lower root osmotic potential than the non-silicon (PEG) plants (**Figure 3A**).

The proline concentration in the roots was not affected by water stress or Si addition on the fifth day. Water stress decreased the proline level, and there was no difference between PEG and PEG + Si treatments (**Figure 3B**).

### Expression of Aquaporin Genes

**FIGURE 4:**
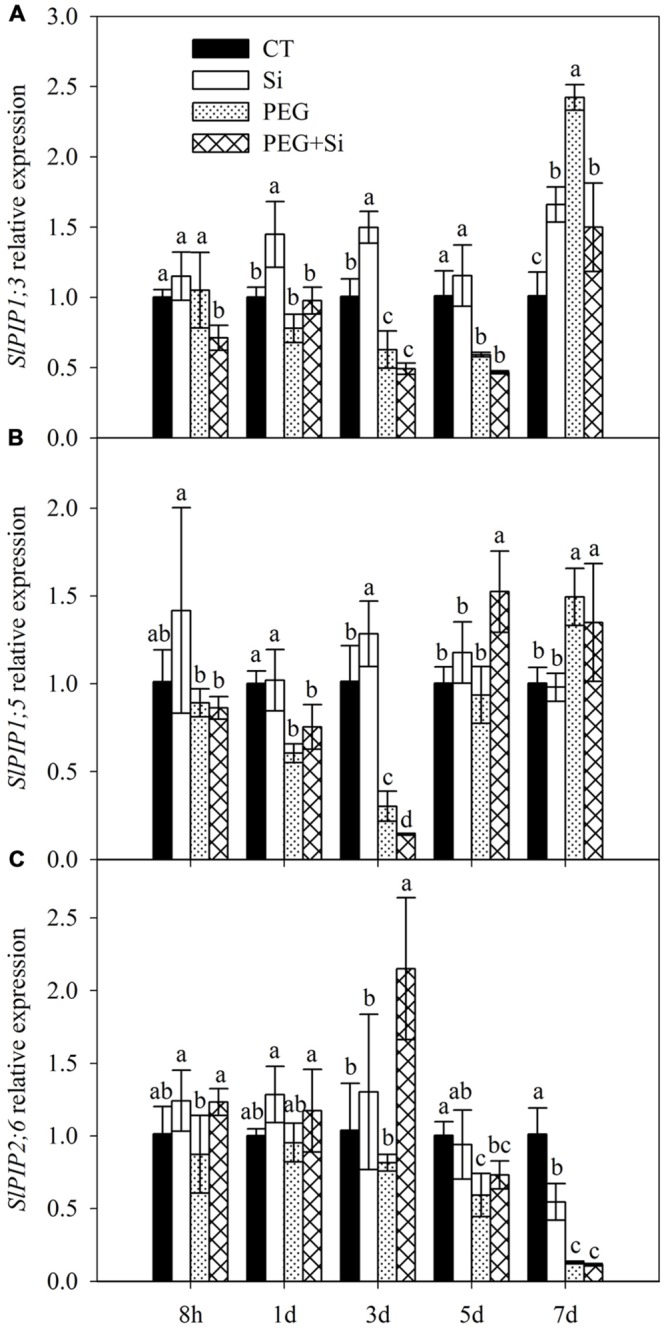
**Effects of silicon on relative transcript levels of main plasma membrane aquaporin genes in the roots of tomato seedlings grown under water stress.** Data are shown as means ± SD (*n* = 5). Bars with the same letters are not significantly different between treatments at each time point at *P* < 0.05. **(A)** *SlPIP1;3* expression; **(B)** *SlPIP1;5* expression; **(C)** *SlPIP2;6* expression.

In our previous transcriptome analysis, we found that *SlPIP1;3, SlPIP1;5*, and *SlPIP2;6* are among the genes that mainly contribute to the total expression of plasma membrane aquaporins (data not shown). Therefore, we determined their expressions in this study. In non-stress conditions, the expression of *SlPIP1;3* was significantly increased by Si addition on the first, third, and seventh days, respectively (**Figure 4A**). The expression of *SlPIP1;5* was only slightly increased by added Si on the third day (**Figure 4B**), whereas the expression of *SlPIP2;6* was decreased by Si addition on the seventh day (**Figure 4C**).

In water stress conditions, Si addition decreased the *SlPIP1;3* expression on the eighth hour and seventh day after stress start, but it did not change the expression at other time points (**Figure 4A**). The expression of *SlPIP1;5* was decreased on the third day but increased on the fifth day (**Figure 4B**). The *SlPIP2;6* expression was increased on the eighth hour and third day, but it was not changed at other time points. By and large, Si addition did not increase the expressions of the three aquaporin genes under water stress (**Figures 4A–C**).

### Relative Electrolyte Leakage and Plasma Membrane Integrity

**FIGURE 5:**
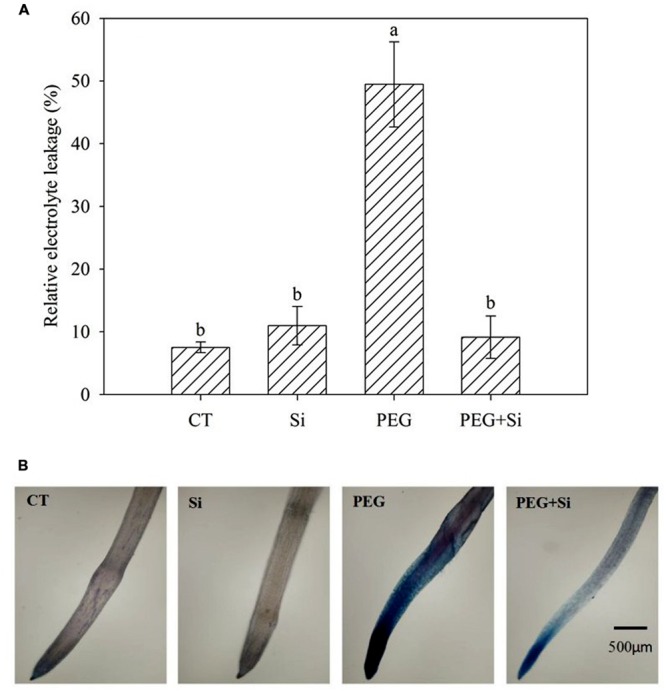
**Effects of silicon on relative electrolyte leakage and plasma membrane integrity of roots in tomato seedlings grown under water stress.** The relative electrolyte leakage of roots was determined after 5 days of water stress (*n* = 4). The histochemical analysis was conducted 6 days after water stress, and the plasma membrane integrity of roots was monitored using the Evan blue staining method. **(A)** Relative electrolyte leakage; **(B)** Plasma membrane integrity.

In non-stress conditions, Si addition did not change the relative electrolyte leakage (**Figure 5A**) and plasma membrane integrity of roots (**Figure 5B**). Water stress dramatically increased the relative electrolyte leakage (**Figure 5A**), and decreased the plasma membrane integrity, as shown by Evans blue staining (**Figure 5B**). Under water stress, Si addition inhibited the increase of relative electrolyte leakage and maintained the plasma membrane integrity (**Figures 5A,B**).

### Malondialdehyde Concentration and Lipid Peroxidation

**FIGURE 6:**
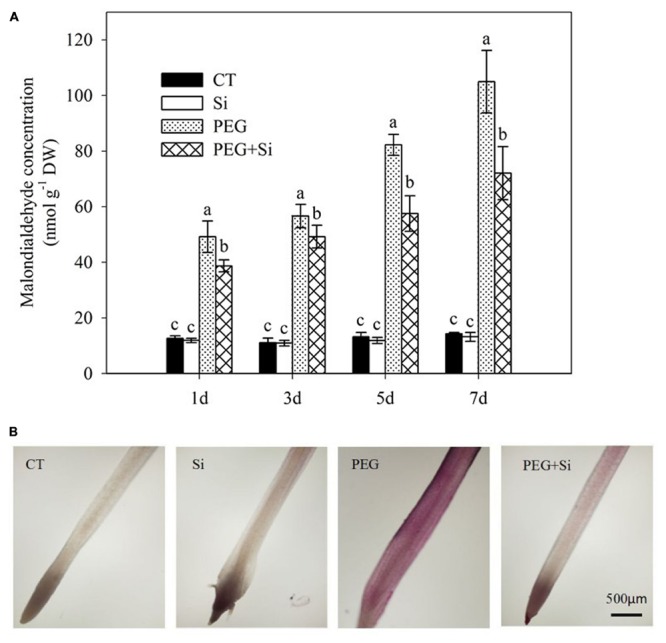
**Effects of silicon on lipid peroxidation of tomato seedlings grown under water stress. (A)** Lipid peroxidation as indicated by malondialdehyde level. Data are means ± SD for malondialdehyde level (*n* = 3). Bars with the same letters are not significantly different at *P* < 0.05. **(B)** Histochemical staining. The histochemical analysis was conducted 6 days after water stress, and the lipid peroxidation in roots was monitored using the Schiff’s reagent staining method.

Under non-stress conditions, added Si had no significant effect on the root malondialdehyde level (**Figure 6A**). The malondialdehyde level was significantly increased under water stress, and it was decreased by added Si (**Figure 6A**). The root lipid peroxidation was monitored by Schiff’s reagent staining. Under water stress, the lipid peroxidation was obviously increased, and it was significantly decreased in Si-supplied plants (**Figure 6B**).

### ROS Level

**FIGURE 7:**
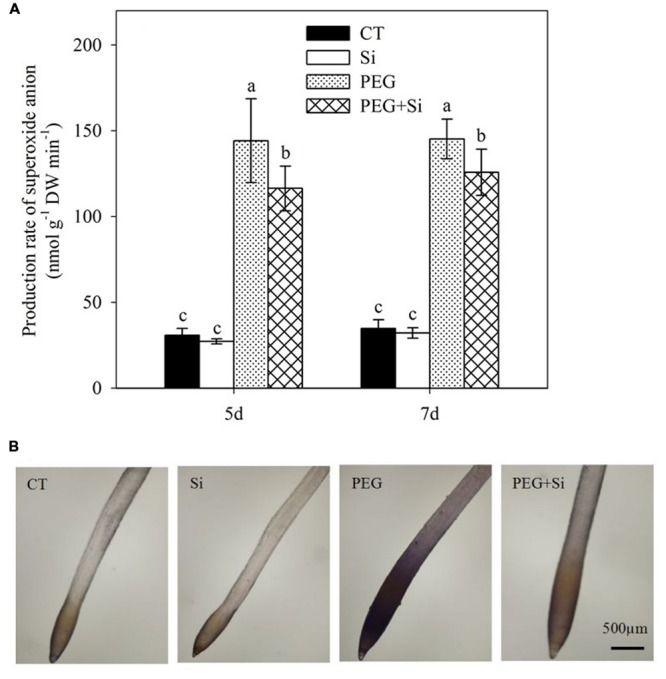
**Effects of silicon on superoxide anion radical ($O_{2}^{•-}$) levels in the roots of tomato seedlings grown under water stress.** Data are shown as means ± SD (*n* = 3) for production rate of superoxide anion radical. Bars with the same letters are not significantly different at *P* < 0.05. The histochemical analysis was conducted 6 days after water stress, and the $O_{2}^{•-}$ production rate was monitored using nitroblue tetrazolium. **(A)** Production rate of superoxide anion; **(B)** Histochemical staining.

Under non-stress conditions, added Si did not change the $O_{2}^{•-}$ production rate in the roots (**Figure 7A**). Water stress obviously increased the $O_{2}^{•-}$ production rate, which was significantly decreased by Si addition (**Figure 7A**). Si-mediated decrease in $O_{2}^{•-}$ production was confirmed by the histochemical staining method using nitroblue tetrazolium (**Figure 7B**).

**FIGURE 8:**
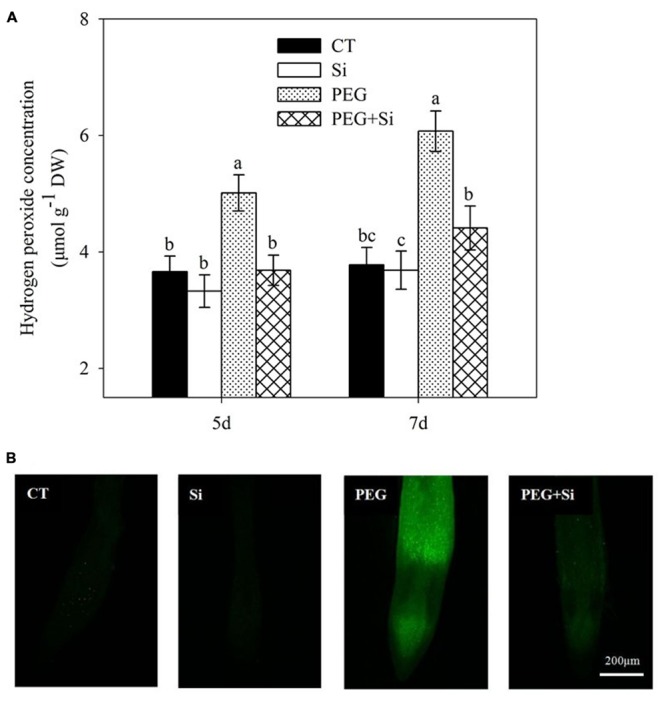
**Effects of silicon on hydrogen peroxide (H_2_O_2_) levels in the leaves of tomato seedlings grown under water stress.** Data are shown as means ± SD (*n* = 3) for H_2_O_2_ level. Bars with the same letters are not significantly different at *P* < 0.05. The histochemical analysis was conducted 6 days after water stress, and the H_2_O_2_ level was monitored using a fluorescent dye 2,7-dichlorofluorescin diacetate. **(A)** Hydrogen peroxide concentration; **(B)** Histochemical staining.

The change of H_2_O_2_ level in roots under water stress in the absence or presence of added Si was similar to that of $O_{2}^{•-}$ production rate, as shown in **Figure 8**.

### Antioxidant Defense

**FIGURE 9:**
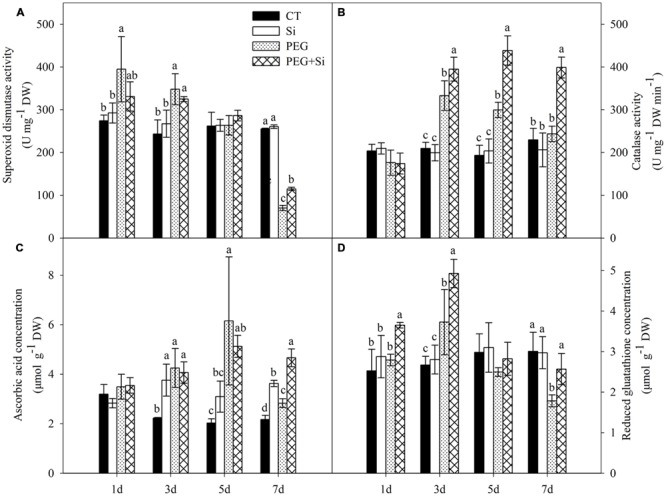
**Effects of silicon on activities of superoxide dismutase and catalase, contents of ascorbic acid and reduced glutathione (GSH) in roots of tomato seedlings grown under water stress.** Data are means ± SD (*n* = 3). Bars with the same letters or without letters are not significantly different between treatments at each time point at *P* < 0.05. **(A)** Superoxide dismutase activity; **(B)** Catalase activity; **(C)** Ascorbic acid concentration; **(D)** Reduced glutathione concentration.

As shown in **Figure 9A**, under non-stress conditions, added Si did not have any significant effect on the SOD activity in the root. Compared with the control, the SOD activity was significantly increased at the early stage of water stress (before 3 days), but it was not changed on the fifth day and decreased on the seventh day under water stress. Under water stress, added Si did not significantly change the SOD activity except on the seventh day, when added Si increased the activity.

Under non-stress conditions, added Si did not change the CAT activity in the root (**Figure 9B**). Under water stress, the CAT activities were increased significantly on the third and fifth day, but they were not changed on the first and seventh day. Si addition did not change the CAT activity on the first day, but it significantly increased the CAT activities from the third day under water stress (**Figure 9B**).

Single Si addition or water stress treatment did not change the root AsA concentration on the first day, but increased the concentration from the third day onward (**Figure 9C**). Under water stress, Si addition only increased the AsA concentration after 7 days of stress treatment (**Figure 9C**).

Under non-stress conditions, Si addition did not affect the level of GSH in the roots (**Figure 9D**). Water stress increased the glutathione concentration on the third day but decreased it on the seventh day. Under water stress, added Si increased the glutathione concentration except on the fifth day (**Figure 9D**).

### Relationship Between Lp and Both Malondialdehyde and ROS Levels

**FIGURE 10:**
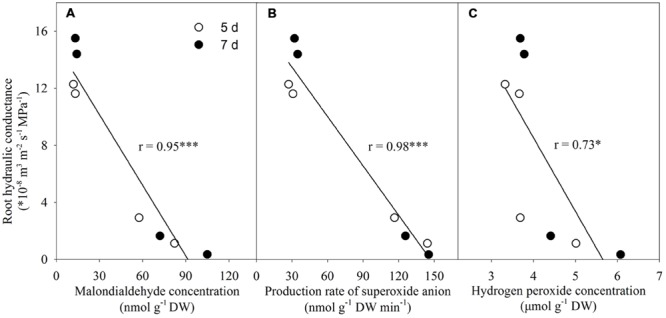
**Correlations between root hydraulic conductance and both malondialdehyde and reactive oxygen species levels.** ^∗^*P* < 0.05, ^∗∗∗^*P* < 0.001. Pearson correlation analysis was conducted using SPSS software (SPSS Inc. 16.0). **(A)** Correlation between root hydraulic conductance and malondialdehyde concentration; **(B)** Correlation between root hydraulic conductance and production rate of superoxide anion; **(C)** Correlation between root hydraulic conductance and hydrogen peroxide concentration.

There was a negative linear correlation between Lp and malondialdehyde concentration (**Figure 10A**). Similar correlations also existed between Lp and both production rate of superoxide anion radical and H_2_O_2_ level (**Figures 10B,C**).

## Discussion

### Si Addition Increased the Tolerance to Water Stress in Tomato Seedlings

Previous research has shown that Si application could enhance drought tolerance of plants (see review by Zhu and Gong, 2014). However, most of the studies have been conducted on Si-accumulating plants, whereas less information is available about the role of Si on drought/water stress tolerance of tomato – a ‘Si excluder’ (Nikolic et al., 2007). In this study, compared with the control, the dry matter accumulation of tomato was decreased under water stress, while it was not significantly changed in the presence of added Si (**Figure 1A**). The growth improvement by added Si corresponded to the maintenance of higher net photosynthetic rate (**Figure 1C**). Our observation together with previous studies suggests that Si can not only improve the drought-tolerance of high Si-accumulating plants, but also improve that of the low Si-accumulating plants, even the so called “Si excluder” like tomato. Our study supports the view that the effect of Si is not proportional to its accumulation in plants, and that low Si accumulation does not mean its low function, as proposed by Katz (2014). Si-mediated improvement in drought tolerance of tomato – non-Si-accumulator suggests that tomato is an ideal model for the biochemical function research of Si. Our results also imply a potential application of Si fertilizer in tomato production in arid or semi-arid regions.

### Si Improved Root Water Uptake in Tomato Under Water Stress

Si-mediated enhancement of leaf photosynthesis under water stress could be attributed to improved plant water status. In this study, the water status of tomato leaves were significantly improved by added Si under water stress. The results are in accordance with those observed in Si-accumulators such as sorghum (Hattori et al., 2005), wheat (Pei et al., 2010), and rice (Ming et al., 2012).

Decrease in leaf transpiration is beneficial to maintain a good leaf water status under the same root water absorption conditions. In early years, it was generally believed that reduction in plant transpiration was a key mechanism of Si-mediated drought tolerance. It was suggested that Si deposition on the leaf surface may decrease the transpiration via cuticle (Savant et al., 1999). Gao et al. (2006) found that application of Si had no significant effects on the cuticular conductance and cuticular transpiration in maize leaves, but significantly reduced the stomatal conductance and stomatal transpiration. In this study, however, the transpirational rate of tomato leaves was increased by Si addition under water stress (**Figure 1D**). Our results are in accordance with those observed in wheat (Gong et al., 2005), sorghum (Hattori et al., 2005), and rice (Chen et al., 2011) under drought stress. However, in cucumber plants, Hattori et al. (2008) observed that Si addition did not affect the leaf transpirational rate under water stress. These studies suggest that the effect of Si on plant transpiration may be related to plant species, and a reduction in transpirational loss of water is not a universal mechanism for Si-mediated improvement of water status in plants. Our results suggest that other factor(s) contributed to Si-mediated improvement in leaf water status in tomato plants.

Leaf water status is determined by water uptake and transport, as well as transpirational loss. In this study, since Si addition did not decrease leaf transpiration of tomato seedlings, but even increased it under water stress conditions (**Figure 1D**), therefore, it is speculated that Si-mediated improvement in leaf water status should be attributed to the enhancement of root uptake and/or transport.

Previous researchers have indicated that Si may affect the cell wall properties of xylem vessels (Gao et al., 2004; Diogo and Wydra, 2007). Si-mediated changes in cell wall properties of xylem vessels may regulate water transport and therefore influence plant water relations. In this study, we did not investigate the effect of Si on water transport in tomato plants. However, Liu et al. (2014) found that Si did not change the sorghum leaf-specific water conductivity – a measure of hydraulic capacity of stem to supply leaves with water. Recently, we observed that Si addition only increased the leaf-specific water conductivity in cucumber after 10 or 15 days of salt stress (75 mM), depending on the cultivars (Zhu et al., 2015). Although the effect of Si on leaf-specific conductivity of the stem was not investigated in this study, previous study suggested that leaf-specific conductivity is not a main restraint factor for water transport under water stress (Liu et al., 2014). Therefore, in this study, the improvement of leaf water status mediated by Si addition in tomato seedlings under water stress might be mainly accounted for by an increase in root water uptake.

Root hydraulic conductance (Lp) is an important parameter that reflects the ability of water uptake (Liu et al., 2014). In the liquid components of soil (growth medium)-plant-air continuum, the Lp is generally the lowest (Vandeleur et al., 2009) and therefore determines water movement rate in the whole plant. In this study, Si addition significantly increased the root hydraulic conductance of tomato, which was dramatically decreased under water stress (**Figure 2**). These results suggest that addition of Si increased the root water uptake ability, and thus improved the water status of tomato plants under water stress.

### Si did not Improve Root Water Uptake by Increasing Osmotic Driving Force, nor by Up-regulating the Transcriptions of Plasma Membrane Aquaporin Genes in Tomato Roots

The size of Lp is associated with root anatomy, water permeability and driving force (Sutka et al., 2011; Liu et al., 2014). In a recent study, Liu et al. (2014) did not observe any Si-mediated changes in vessel diameter or vessel number. In this work, we did not investigate the effect of Si on the root anatomic characteristics in tomato, but we did not observe any change in root mean diameter as a result of Si addition, irrespective of water stress (data not shown). In addition, the root/shoot ratio was also not changed by Si addition under water stress (**Figure 1B**). These results suggest that the Si-mediated increase in Lp under water stress may not be mainly attributed to the change of root anatomy.

Water movements in the roots include two aspects: a radial movement from the root surface to xylem vessels, and an axial (longitudinal) movement along the xylem vessels. The hydraulic resistance is usually much higher in the former than in the latter (Steudle and Peterson, 1998). In the radial movement, water moves along three pathways: the apoplastic, symplastic, and transcellular pathways. The latter two are collectively referred to as ‘cell-to-cell’ pathway (Chaumont and Tyerman, 2014). Under water stress, the hydraulic resistance of apoplastic pathway is very high and therefore less used by plants, while the cell-to-cell pathway plays a more important role in water uptake (Javot and Maurel, 2002). In the cell-to-cell pathway, osmotic gradient between growth medium and root inside is beneficial for water uptake (Javot and Maurel, 2002). Under stress conditions, the accumulation of compatible organic solutes (such as proline) may decrease the cell osmotic potential and therefore forms an osmotic gradient (Ashraf and Foolad, 2007). In this study, compared with non-stress treatment, water stress did not change the root osmotic potential in Si-treated plants (**Figure 3A**); moreover, no proline accumulation was observed under water stress (**Figure 3B**). These results suggest that Si-mediated increase in root water uptake was not due to an increase in osmotic driving force in tomato under water stress. Similar results were also observed in sorghum (Liu et al., 2014). However, Zhu et al. (2015) found that, under salt stress, Si addition induced an accumulation of soluble sugar and a decrease of root xylem osmotic potential in cucumber cv. ‘JinYou 1,’ but such effects were not observed in cv. ‘JinChun 5.’ Therefore, under stress conditions, the role of osmotic driving force in Si-mediated enhancement of water uptake is genotype-dependent.

In the cell-to-cell pathway, water move is mainly controlled by the amount and activity of aquaporins (Vandeleur et al., 2009). In this work, we analyzed the expressions of *SlPIP1;3, SlPIP1;5*, and *SlPIP2;6* (**Figure 4**), which were the three plasma membrane aquaporin genes that mainly contributed to the total expression according to our previous RNA-Seq data (data not shown). However, by and large, the expressions of these aquaporin genes were not significantly changed (less than twofold) as a result of Si addition under water stress (**Figure 4**). Our results are in contrast to those observed in sorghum by Liu et al. (2014), who found that Si-pretreatment significantly increased the aquaporin expressions under water stress. Recently, Liu et al. (2015) and Zhu et al. (2015) also observed that Si addition increased the aquaporin expressions in sorghum and cucumber under salt stress, respectively. The reasons for the differences in Si effects on aquaporin gene expressions under stress conditions are not clear, but may be related to plant species. Since control of protein levels can be transcriptional or posttranscriptional, protein levels may be different from mRNA level. Further work is needed to investigate the expressions of plasma membrane aquaporins at protein level. Despite of this, our results suggest that Si-mediated up-regulation of aquaporin gene expression at transcriptional level is not a general mechanism for the enhancement of root hydraulic conductance under stress conditions.

### Si Might Improve Root Hydraulic Conductance by Decreasing ROS Production and Membrane Damage in Tomato Roots

In plant cells, the scavenging of ROS is by the antioxidant defense system, which includes both enzymatic antioxidants (such as SOD, CAT and ascorbate peroxidase) and non-enzymatic antioxidants (such as AsA and GSH; Gong et al., 2005; Shi et al., 2014). Under stress conditions, the balance between ROS production and scavenging is usually broken, leading to an excess accumulation of ROS (Gong et al., 2005), which induces oxidative stress. Oxidative stress causes plasma membrane injury and affects root hydraulic conductance. Benabdellah et al. (2009) observed that high concentrations of exogenous H_2_O_2_ affected the root hydraulic conductance, and the changes of hydraulic conductance were inversely matched with the changes of membrane electrolyte leakage and ROS level. In an earlier study, Aroca et al. (2005) suggested that, compared with the chilling-sensitive maize genotype, the tolerant genotype had better ability to avoid or repair membrane damage, therefore its root hydraulic conductance could recover from initial drop under chilling stress. These studies suggest that avoidance of membrane damage is important to maintain root hydraulic conductance. The positive effect of silicon on ROS detoxification in tomato plants has recently been observed in salt stress conditions (Muneer and Jeong, 2015), while little information is available under drought/water stress. In this work, Si addition inhibited ROS overproduction (**Figures 7A,B** and **8A,B**), and thus decreased membrane lipid peroxidation (**Figures 6A,B**) and improved plasma membrane integrity (**Figure 5B**) in tomato seedlings under water stress. The Si-mediated alleviation of oxidative damage under water stress corresponded with the increase in antioxidant defense (**Figure 9**): on day 1, the GSH level was increased by Si addition; on day 3, both the GSH level and CAT activity were increased; on day 5, the CAT activity was increased; on day 7, the activities of SOD and CAT, and the concentrations of AsA and GSH were all increased by Si addition under water stress. In this study, added Si mediated an increase in root hydraulic conductance under water stress (**Figure 2**), which corresponded with the decreased membrane damage (**Figures 5** and **6**). Negative correlations between the root hydraulic conductance and the levels of both ROS and lipid peroxidation product – malondialdehyde was also observed (**Figure 10**). These results suggest that Si-mediated decrease in oxidative damage of membrane might have contributed to the increase in root hydraulic conductance.

It is not clear how oxidative damage exactly affects the root hydraulic conductance. It is reasonable to speculate that oxidative damage causes plasma membrane dysfunction and may thus affect the function of plasma membrane aquaporins, which play an important role in root water uptake, especially under water stress (Vandeleur et al., 2009; Liu et al., 2014). On the other hand, the overproduced ROS under water stress may negatively regulate the activities of plasma membrane aquaporins. Ye and Steudle (2006) observed the oxidative gating of aquaporins in maize roots. Boursiac et al. (2008a) suggested that ROS did not gate aquaporins through a direct oxidative mechanism, but might act through some signaling mechanism in *Arabidopsis* roots. They found that H_2_O_2_ induced the internalization of plasma membrane aquaporins under salt stress. Their further study (Boursiac et al., 2008b) demonstrated that ROS-dependent signaling mechanism regulated the aquaporin phosphorylation status and thus its intracellular trafficking.

In addition, H_2_O_2_ is involved in the formation of suberin lamellae (Razem and Bernards, 2002). In endodermis and exodermis of roots, suberin forms a hydrophobic barrier (Enstone et al., 2003). Hence, in this study, there is a possibility that the stressed roots with added Si developed less suberin lamellae than those without added Si, and therefore had higher water permeability. Fleck et al. (2011) observed that Si enhanced suberization and lignification in the roots of rice. However, whether Si enhances suberization in tomato roots remains unclear. The effects of Si and H_2_O_2_ on the formation of suberin lamellae also need to be investigated under water stress conditions.

Despite of the complex mechanisms for the actions of both membrane oxidative damage and ROS accumulation on root hydraulic conductance, Si-mediated alleviation of oxidative damage and decrease in ROS level in the roots may have contributed to the increase of root hydraulic conductance and facilitated root water uptake in tomato seedlings (**Figure 10**).

## Conclusion

Si can increase water stress tolerance of tomato – a ‘Si excluder.’ Si enhances water stress tolerance via enhancing root hydraulic conductance and water uptake. Si-mediated decrease in membrane oxidative damage via enhanced antioxidant defense may have contributed to the enhanced root hydraulic conductance. Further studies are needed to explore, how Si triggers the antioxidant defense in tomato plants under water stress.

## Author Contributions

Performed the experiments: YS, YZ, WH, RF, YH, and JG. Analyzed the data: YS and YZ. Draft the paper: YS. Conceived and designed the experiments, and revise the paper: HG.

## Conflict of Interest Statement

The authors declare that the research was conducted in the absence of any commercial or financial relationships that could be construed as a potential conflict of interest.
